# Caffeamide 36-13 Regulates the Antidiabetic and Hypolipidemic Signs of High-Fat-Fed Mice on Glucose Transporter 4, AMPK Phosphorylation, and Regulated Hepatic Glucose Production

**DOI:** 10.1155/2014/821569

**Published:** 2014-07-20

**Authors:** Yueh-Hsiung Kuo, Cheng-Hsiu Lin, Chun-Ching Shih

**Affiliations:** ^1^Department of Chinese Pharmaceutical Sciences and Chinese Medicine Resources, China Medical University, Taichung City 40402, Taiwan; ^2^Department of Biotechnology, Asia University, Taichung City 41354, Taiwan; ^3^Department of Internal Medicine, Fengyuan Hospital, Ministry of Health and Welfare, Fengyuan District, Taichung City 42055, Taiwan; ^4^Graduate Institute of Pharmaceutical Science and Technology, College of Health Science, Central Taiwan University of Science and Technology, No. 666 Buzih Road, Beitun District, Taichung City 40601, Taiwan

## Abstract

This study was to investigate the antidiabetic and antihyperlipidemic effects of (E)-3-[3, 4-dihydroxyphenyl-1-(piperidin-1-yl)prop-2-en-1-one] (**36-13**) (TS), one of caffeic acid amide derivatives, on high-fat (HF-) fed mice. The C57BL/6J mice were randomly divided into the control (CON) group and the experimental group, which was firstly fed a HF diet for 8 weeks. Then, the HF group was subdivided into four groups and was given TS orally (including two doses) or rosiglitazone (Rosi) or vehicle for 4 weeks. Blood, skeletal muscle, and tissues were examined by measuring glycaemia and dyslipidemia-associated events. TS effectively prevented HF diet-induced increases in the levels of blood glucose, triglyceride, insulin, leptin, and free fatty acid (FFA) and weights of visceral fa; moreover, adipocytes in the visceral depots showed a reduction in size. TS treatment significantly increased the protein contents of glucose transporter 4 (GLUT4) in skeletal muscle; TS also significantly enhanced Akt phosphorylation in liver, whereas it reduced the expressions of phosphoenolpyruvate carboxykinase (PEPCK) and glucose-6-phosphatase (G6Pase). Moreover, TS enhanced phosphorylation of AMP-activated protein kinase (phospho-AMPK) both in skeletal muscle and liver tissue. Therefore, it is possible that the activation of AMPK by TS resulted in enhanced glucose uptake in skeletal muscle, contrasting with diminished gluconeogenesis in liver. TS exhibits hypolipidemic effect by decreasing the expressions of fatty acid synthase (FAS). Thus, antidiabetic properties of TS occurred as a result of decreased hepatic glucose production by PEPCK and G6Pase downregulation and improved insulin sensitization. Thus, amelioration of diabetic and dyslipidemic state by TS in HF-fed mice occurred by regulation of GLUT4, G6Pase, and FAS and phosphorylation of AMPK.

## 1. Introduction

Diabetes mellitus (DM) is a metabolic disease characterized by chronic hyperglycemia accompanied by many metabolic disorders associated with hyperlipidemia, obesity, and coronary heart disease. Type 2 diabetes mellitus (T2D) accounts for 90% to 95% of all patients [1] and is caused by the combination of *β*-cell dysfunction and insulin resistance. Insulin resistance is characterized by a decrease in insulin-stimulate glucose uptake in skeletal muscle via glucose transporter 4 (GLUT4) and by impaired suppression of glucose production in liver [2]. Both genetic (heredity) and environmental factors (obesity and leisure life style) play an important role in T2D. The C57BL/6J mice have shown to be susceptible to high-fat (HF) diet-induced hyperlipidemia, obesity, and type 2 diabetes [3].

**Figure 1:**
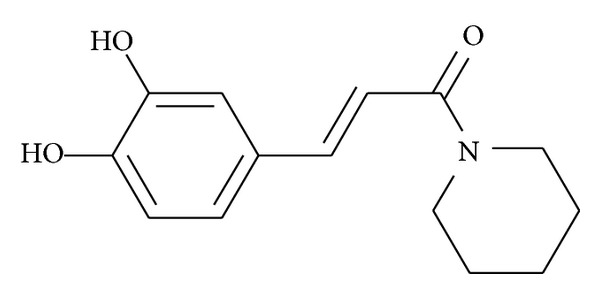
Structure of caffeamide** 36-13 **(TS).

Various natural polyphenolic compounds are demonstrated to exert anti-inflammatory, antioxidant, anticarcinogenic, and cardiovascular protective effects [4–6]. Resveratrol and curcumin are successfully employed in the prevention of a variety of metabolic disorders [7, 8]. (*E*)-3-[3, 4-dihydroxyphenyl-1-(piperidin-1-yl)prop-2-en-1-one]** 36-13** (Caffeamide 36-13; TS) (Figure 1) is one of caffeic acid amide derivatives and structurally similar to caffeic acid phenylethyl amide (CAPA) and resveratrol. Several bioactive properties of similar compounds have been shown with potent antidiabetic activity. Caffeic acid and caffeic acid phenyl ester (CAPE) are widely distributed in the plant kingdom. CAPE is known to stimulate glucose uptake through AMP-activated protein kinase (AMPK) activation in skeletal muscle cells and improve insulin resistance [9]. One of caffeamide derivatives, KS370G, has been shown to exert hypoglycemic activity through insulin-dependent and insulin-independent mechanism [10]. Moreover, CAPA was demonstrated to exhibit hypoglycemic properties [11]. Since amide is more resistance to esterase, it is foreseeable that TS is more stable than CAPE in vivo. The main difference between TS and KS370G compound is that the structure of TS is a piperidine ring. There are no reports about TS describing health benefit of human disorders; thus this study was designed to determine whether TS could ameliorate diabetes and hyperlipidaemia in high-fat-fed mice.

GLUT4 is the major insulin-regulated glucose transporter expressed mainly in skeletal muscle [12, 13]. Insulin stimulates glucose uptake in these cells primarily by inducing net translocation of GLUT4 from the intracellular storage sites to the plasma membrane [14]. Impairment of GLUT4 expressions and GLUT4 translocation and/or insulin signaling may affect insulin-stimulated glucose uptake, and that would result in insulin resistance and hyperglycemia [15, 16]. Therefore, the improvements of GLUT4 contents and/or translocation to the plasma membrane have long been regarded as a potential target in the treatment of diabetes mellitus. In addition, Akt activation in liver contributes to the insulin-mediated suppression of gluconeogenesis with an associated downregulation of phosphoenolpyruvate carboxykinase (PEPCK) and glucose-6-phosphatase (G6Pase) [17].

AMPK is a major cellular regulator of lipid and glucose metabolism. The identification of AMPK phosphorylation as a likely mechanism is particularly interesting in relation to diabetes and obesity because activation of AMPK inhibits lipid synthesis and can improve insulin action [18, 19]. Based on a number of studies showing that AMPK regulates a variety of different metabolic disorders, it is widely recognized as a useful and safe target for the treatment of metabolic disorders such as T2D and dyslipidemia [18, 19]. Since lipid and glucose metabolism is dysregulated in type 2 diabetes mellitus, AMPK modulators have been suggested to be promising therapies.

Since activation of AMPK results in increased lipid and glucose catabolism [20], the effect of TS on AMPK activity, GLUT4 protein content, and hepatic Akt activation is investigated in HF-fed mice. Phosphorylation of Thr 172 of *α* subunits is essential for AMPK activity [21]. As one of the possible mechanisms of action, this study also examined its effect on the expression of genes involved in antidiabetes and lipogenesis in the liver tissue, including PEPCK, G6Pase, and fatty acid synthase (FAS).

## 2. Materials and Methods

### 2.1. Preparation of Caffeamide 36-13

Dissolve a 100 mg caffeic acid in 1 mL* N*,*N-*dimethylformamide and 1 mol and 0.08 mL triethylamine in a two-neck bottle and then add into 1.2 mol, 5 mL dichloromethane (CH_2_Cl_2_) containing the piperidine, and 1.2 mol benzotriazol-1-yloxytris(dimethylamino)phosphonium hexafluorophosphate (BOP) to react for 30 minutes at 0°C, followed by reacting for 2 hours at the room temperature. After the reaction is finished, remove CH_2_Cl_2_. The resulting solution is then added into 50 mL water, which is extracted by the ethyl ester. Collect the organic phase, and wash the organic phase with 1 mol HCL, 1 mol NaHCO_3_, and water, wherein the organic phase is further recollected, followed by removing water therefrom by MgSO_4_, performing a filtration, a condensation, and a column chromatography. Therefore, the product, (*E*)-3-[3, 4-dihydroxyphenyl-1-(piperidin-1-yl)prop-2-en-1-one] (Figure 1), was obtained in 75% yield. White solid; mp: 144–146°C; IR *ν*_max⁡  _ (cm^−1^): 3431, 3115, 3061, 1651, 1618, 1593, 1506, 1423, 1278, 1244, 1045; ^1^H NMR (CD_3_COCD_3_, 400 MHz): *δ* 1.54–1.64 (6 H, m), 3.53 (2 H, t, *J* = 6.8 Hz), 3.66 (2 H, t, *J* = 6.8 Hz), 6.82 (1 H, d, *J* = 8.2 Hz), 6.96, 7.43 (each 1 H, d, *J* = 15.2 Hz), 7.01 (1 H, dd, *J* = 8.2, 1.6 Hz), 7.14 (1 H, d, *J* = 1.6 Hz); UV (MeOH) *λ*_max⁡  _ (log *ε*): 325(4.32), 291(4.19), 2.30(4.26), 2.19(4.50) nm; HRESIMS* m/z*: 270.1106 [M+ Na]^+^(calcd. for C_14_H_17_NO_3_Na, 270.1109).

### 2.2. Animals and Experimental Design

All animal procedures were performed as per guidelines provided by the Institutional Animal Care and Use Committee of Central Taiwan University of Science and Technology. The study contained two parts including part 1: oral glucose tolerance test (OGTT). The ICR mice normal mice (*n* = 5) were fasted for 12 h but were allowed access to 40 mg/kg TS or an equivalent amount of normal vehicle (water) was given orally 30 min before an oral glucose load (1 g/kg body weight). Blood samples were collected from the retro-orbital sinus of fasting mice at the time of the glucose administration (0) and every 30 minutes until 3 hours after glucose administration to determine the levels of glucose. The part 2 animal study, C57BL/6J mice (4-5 weeks old), were purchased from the National Laboratory Animal Breeding and Research Center, National Science Council. Animals were maintained on a 12 h light/dark cycle (light cycle: 7 a.m. to 7 p.m.). Seven days after acclimation, the C57BL/6J mice were divided randomly into two groups. The control (CON) group (*n* = 9) was fed low-fat diet (Diet 12450B, Research Diets, Inc., New Brunswick, NJ 08901, USA), whereas the experimental group (*n* = 36) was fed a 45% high-fat diet (Diet 12451, Research Diets, Inc., New Brunswick, NJ 08901, USA) for 12 weeks. The low-fat diet was composed of protein 20%, carbohydrate 70%, and fat 10%, whereas high-fat diet was composed of protein 20%, carbohydrate 35%, and fat 45% (of total energy, % kcal). After 8-week diet-induction period, the high-fat treated mice were randomly subdivided into four groups (*n* = 9 per group). TS (including T1: 10 and T2: 20 mg/kg/day) or rosiglitazone (Rosi; 1% methylcellulose 10 mg/kg body weight, obtained from GlaxoSmithKline product number: BRL49653 C) was administrated through oral gavage one time per day from 9 to 12 week of the experiment, and the mice were still on the high-fat diet, while the CON and high-fat control (HF) mice were treated with vehicle only. The body weight was measured weekly throughout the study. The compositions of the experimental diets are shown as described [22]. At the end of the study, we deprive food from animal (from 10 p.m. to 10 a.m.). The next day (the 85th day), the mice were sacrificed for blood and tissue collection and analysis. The mice were untreated with TS or Rosi at the 85th day. Livers and white adipose tissues (WATs) (including epididymal, mesenteric, and retroperitoneal WAT) were excised according to the defined anatomical landmarks, and the weights of tissues were measured. Tissues were immediately frozen using liquid nitrogen and then kept at −80°C for the analysis of target gene expression. Heparin (30 units/mL) (Sigma) was added into blood sample. Plasma samples were collected by centrifugation at 1600 ×g for 15 min at 4°C. The separation of the plasma was finished within 30 min. Plasma were obtained for insulin and leptin assay.

### 2.3. Body Weight, Body Weight Gain, and Food Intake Assay

Body weight and food intake were monitored. Body weight was measured daily at the same time throughout the study. The differences between the body weight of the next day and the former day is defined as body weight gain. The pellet food was weighed and followed by being placed in the cage food container. After 24 h, the remaining food was weighed, and the difference represented the daily food intake. Unconsumed pellet HF food was discarded each day and fresh pellet high-fat diet was provided to ensure consistent food quality throughout the study. The HF food was stored at 4°C.

### 2.4. Blood Parameters Assay

Blood samples (0.8 mL) were collected from the retro-orbital sinus of fasting mice and the level of glucose was measured by the glucose oxidase method (Model 1500; Sidekick Glucose Analyzer; YSI Incorporated, Yellow Springs, OH, USA). Plasma triglycerides (TG), total cholesterol (TC), and free fatty acids (FFA) were analyzed using commercial assay kits according to the manufacturer's directions (Triglycerides-E test, Cholesterol-E test, and FFA-C test, Wako Pure Chemical, Osaka, Japan).

### 2.5. Measurement of Adipocytokine and Insulin Levels in Blood

The levels of adiponectin, leptin, and insulin in blood were analyzed by ELISA using a commercial assay kit according to manufacturer's directions (mouse/rat adiponectin ELISA kit, B-Bridge International, GmbH, Germany; mouse insulin ELISA kit, Shibayagi, Gunma, Japan; and mouse leptin ELISA kit, Morinaga, Yokohama, Japan).

### 2.6. Histopathology of Adipose and Liver Tissue

Small pieces of epididymal WAT and liver tissue were fixed with formalin (200 g/kg) neutral buffered solution and embedded in paraffin. Sections (8 *μ*m) were cut and stained with hematoxylin and eosin. For microscopic examination, a microscope (Leica, DM2500) was used, and the images were taken using a Leica Digital camera (DFC-425-C).

### 2.7. Measurement of Hepatic Lipids

Hepatic lipids were extracted using a previously described protocol [23]. For the hepatic lipid extraction, the 0.375 g liver samples were homogenized with 1 mL distill water for 5 min. Finally, the dried pellet was resuspended in 0.5 mL ethanol and analyzed using a triglycerides kit as used for serum lipids.

### 2.8. Isolation of RNA and Relative Quantization of mRNA Indicating Gene Expression

Total RNA from liver tissue was isolated with a Trizol Reagent (Molecular Research Center, Inc., Cincinnati, OH) according to the manufacturer's directions. The integrity of the extracted total RNA was examined by 2% agarose gel electrophoresis, and the RNA concentration was determined by the ultraviolet (UV) light absorbency at 260 nm and 280 nm (Spectrophotometer U-2800A, Hitachi). The quality of the RNA was confirmed by ethidium bromide staining of 18S and 28S ribosomal RNA after electrophoresis on 2% agarose gel containing 6% formaldehyde. Total RNA (1 *μ*g) was reverse transcribed to cDNA in a reaction mixture containing buffer, 2.5 mM dNTP (Gibco-BRL, Grand Island, NY), 1 mM of the oligo (dT) primer, 50 mM dithiothreitol, 40 U Rnase inhibitor (Gibco-BRL, Grand Island, NY), and 5 *μ*L Moloney murine leukemia virus reverse transcriptase (Epicentre, Madison, WI, USA) at 37°C for 1 h and then heated at 90°C for 5 min to terminate the reaction. The polymerase chain reaction (PCR) was performed in a final 25 *μ*L containing 1 U Blend Taq-Plus (TOYOBO, Japan), 1 *μ*L of the RT first-strand cDNA product, 10 *μ*M of each forward (F) and reverse (R) primer, 75 mM Tris-HCl (pH 8.3) containing 1 mg/L Tween 20, 2.5 mM dNTP, and 2 mM MgCl_2_. Preliminary experiments were carried out with various cycles to determine the nonsaturating conditions of the PCR amplification for all the genes studied. The primers are shown in Table 1. The products were run on 2% agarose gels and stained with ethidium bromide. The relative density of the band was evaluated using AlphaDigiDoc 1201 software (Alpha Innotech Co., San Leandro, CA, USA). All the measured PCR products were normalized to the amount of cDNA of GAPDH in each sample.

### 2.9. Western Immunoblotting Analysis

Protein extractions and immunoblots for the determination of GLUT4, phospho-AMPK (Thr172), and phospho-Akt (Ser 473) proteins were carried out on frozen skeletal muscle and liver tissue from mice according to a previous report [24]. Briefly, samples (0.1 g) were powdered under liquid nitrogen and homogenized for 20 s in 500 *μ*L buffer containing 20 mM Tris-HCl (pH 7.4 at 4°C), 2% SDS, 5 mM EDTA, 5 mM EGTA, 1 mM DTT, 100 mM NaF, 2 mM sodium vanadate, 0.5 mM phenylmethylsulfonyl fluoride, 10 *μ*g/mL leupeptin, and 10 *μ*L/mL pepstatin. 40 *μ*g of each homogenate was mixed with an equal amount of 2x standard SDS sample loading buffer containing 125 mM Tris-HCl (pH 6.8), 4% SDS, 20% glycerol, 10% *β*-mercaptoethanol, and 0.25% bromophenol blue and boiled for 10 min before electrophoresis. The protein contents of GLUT4 (Santa Cruz Biotechnology, CA, USA), phospho-AMPK (Abcam Inc., Cambridge, MA, USA), and phospho-Akt (Cell signaling Technology, Inc., Danvers, MA, USA) were detected by immunoblotting using a rabbit polyclonal antibody. About 0.1 g of liver tissue and skeletal muscle of mice (*n* = 9) was used for the homogenate samples containing lysis buffer (pH6.4) and protease inhibitors. The protein concentration in supernatant was determined with a BCA protein assay kit (Thermo Scientific, Rockford, IL, USA). Twenty micrograms of proteins were separated by electrophoresis on a polyacrylamide gel 10% (SDS-PAGE) and transferred to a nitrocellulose membrane. The membranes were blocked with 5% slim milk in Tris-buffered saline (TBS) (Amersham BioSciences, Uppsala, Sweden) containing 0.05% Tween-20 (Bio Rad, CA, USA) and incubated overnight at 4°C with anti-GLUT4, anti-phospho-AMPK, and anti-phospho-Akt at 1 : 200 dilution. Subsequently, the membranes were washed three times with TBS containing 0.05% Tween-20 and incubated with secondary antibody anti-rabbit (1 : 1000) (Jackson ImmunoResearch Laboratories, Inc., PA, USA) for 1 h. Immunoreactive bands were detected with ECL reagent kit (GE Healthcare BioSciences, Buckinghamshire, UK). The density blotting was analyzed using Alpha Easy FC software (Alpha Innotech Corporation, Randburg, South Africa). Structural proteins GAPDH (Santa Cruz Biotechnology, CA, USA) and *β*-actin (Santa Cruz Biotechnology, CA, USA) were obtained by stripping the nitrocellulose membrane proteins of liver and skeletal muscle.

### 2.10. Statistical Analysis

Data were expressed as mean ± S.E. values. Whenever possible, data were subjected to analysis of variance, followed by Dunnett's multiple range tests, using SPSS software (SPSS Inc., Chicago, IL, USA). *P* < 0.05 was considered to be statistically significant.

## 3. Results

### 3.1. Oral Glucose Tolerance Test (OGTT)

**Figure 2:**
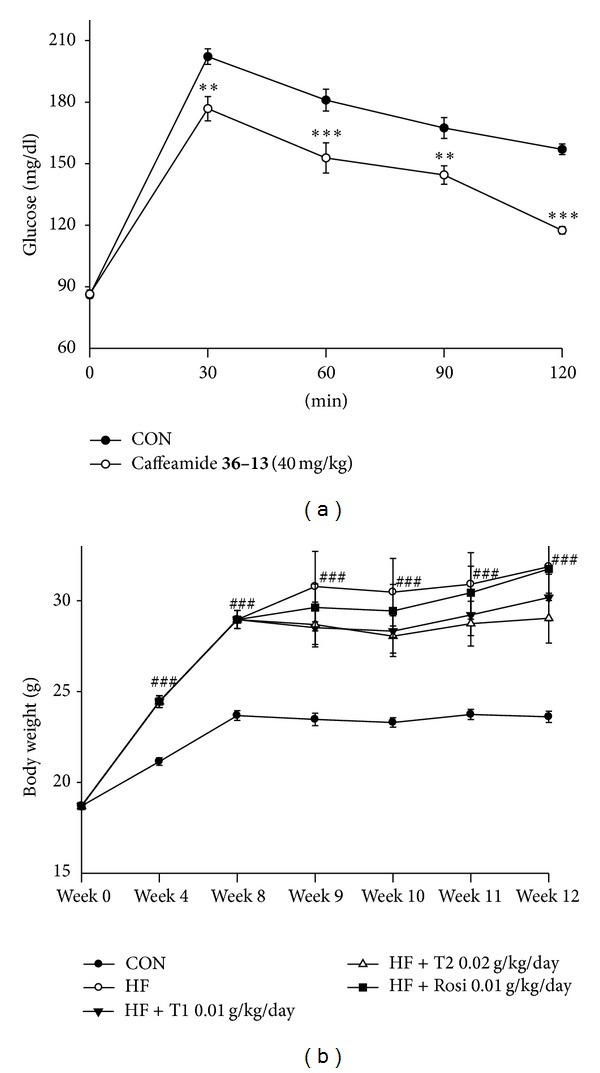
(a) Effects of caffeamide** 36-13 **(TS) on oral glucose tolerance in normal mice. Animals in all groups received oral glucose 30 minutes after TS administration. Blood samples were collected and centrifuged at 3000** **rpm for 10 minutes. Each point is the mean ± S.E. of 5 separate mice. **P < 0.01, ***P < 0.001 significantly different compared with the control group in the same time by ANOVA. (b) Effects of caffeamide** 36-13 **(TS) on body weight. Mice were fed with 45% high-fat diet (HF) or low-fat diet (CON) for 12 weeks. After 8 weeks, the HF mice were treated with vehicle or TS or rosiglitazone (Rosi) accompanied with HF diet for 4 weeks. All values are means ± S.E. (*n* = 9). ^###^ *P* < 0.001 compared with the control (CON) group by ANOVA. TS:** **T1:10, T2:** **20** **mg/kg bodyweight; Rosi: rosiglitazone (0.01** **g/kg body weight). WAT, white adipose tissue; epididymal WAT + retroperitoneal WAT, visceral fat.

The effect of TS on OGTT is shown in Figure 2(a). TS (40 mg/kg) significantly decreased the levels of blood glucose at times ranging from 30 to 120 min glucose-loading when compared with the control in ICR mice.

### 3.2. Body Weight, Body Weight Gain, Food Intake, and Tissue Weight

All group mice started with similar mean body weights (17.6 ± 0.2 g). At week 12, the body weight of all the high-fat diet treated mice is significantly greater than the CON group (*P* < 0.001). Treatment with T1 and T2 showed no significant differences in body weight compared with the vehicle-treated HF group (Figure 2(b)). At week 12, the body weight gain of the HF group is greater than the CON group (*P* < 0.01). The T2-treated group decreased in body weight gain compared with the HF group (*P* < 0.05) (Table 2). At week 12, the vehicle-treated HF group is significantly lower than the CON group in the 4-week food intake (g/day/mice) (*P* < 0.001). Following T1 and T2 treatment, there is no significant difference in food intake (g/day/mice) (Table 2). At week 12, the weights of absolute adipose tissue (epididymal, visceral fat, mesenteric, and retroperitoneal WAT) were markedly greater in the HF group than in the CON group (*P* < 0.001, *P* < 0.001, *P* < 0.001, and *P* < 0.001, resp.). The T1-, T2-, and Rosi-treated groups showed a significant decrease in the weights of absolute epididymal WAT, visceral fat, mesenteric, and retroperitoneal WAT compared with the HF group. No significant difference in the weights of liver and spleen was observed in T1-, T2-, and Rosi-treated groups compared with the HF group. The weights of brown adipose tissue (BAT) were decreased in T2-treated mice as compared with the HF group (*P* < 0.05) (Table 2).

### 3.3. Plasma Glucose Levels

At the beginning of the study, all mice started with similar levels. At weeks 8 and 12, the glucose levels of the HF group were significantly greater than the CON group (*P* < 0.001, *P* < 0.001, resp.). Treatment with T1, T2, and Rosi showed a significant reduction in plasma glucose compared with the HF group (*P* < 0.01, *P* < 0.01, and *P* < 0.001, resp.) (Table 2).

### 3.4. Plasma and Hepatic Lipid

As time passed, the hypercholesterolemia phenomenon was evident for the HF diet. As shown in Table 2, at week 12, the levels of TC, TG, and FFA were greater in the HF group than in the CON group (*P* < 0.001, *P* < 0.001, and *P* < 0.01, resp.). The T1-, T2-, and Rosi-treated groups suppressed the HF diet-induced increases in the concentrations of TG (*P* < 0.01, *P* < 0.001, and *P* < 0.05, resp.). No significant difference in the concentrations of TC was observed in T1-, T2-, and Rosi-treated groups compared with the HF group. Treatment with T1, T2, and Rosi suppressed the high-fat diet-induced increases in the concentrations of FFA (*P* < 0.01, *P* < 0.001, and *P* < 0.05, resp.).

### 3.5. Leptin, Insulin, and Adiponectin Concentration

As shown in Table 2, at week 12, the concentrations of leptin and insulin were greater in the HF group than in the CON group (*P* < 0.001, *P* < 0.001, resp.). Following treatment, T1-, T2-, and Rosi-treated groups significantly decreased leptin levels (*P* < 0.001, *P* < 0.001, and *P* < 0.001, resp.). T1, T2-, and Rosi-treated groups significantly decreased the levels of insulin compared with the HF group (*P* < 0.05, *P* < 0.01, and *P* < 0.05, resp.). The concentrations of adiponectin were lower in the HF group than in the CON group (*P* < 0.01). Following treatment, T1-, T2-, and Rosi-treated groups increased adiponectin levels compared with the HF group.

### 3.6. Histopathology of Adipose and Liver Tissue

**Figure 3:**
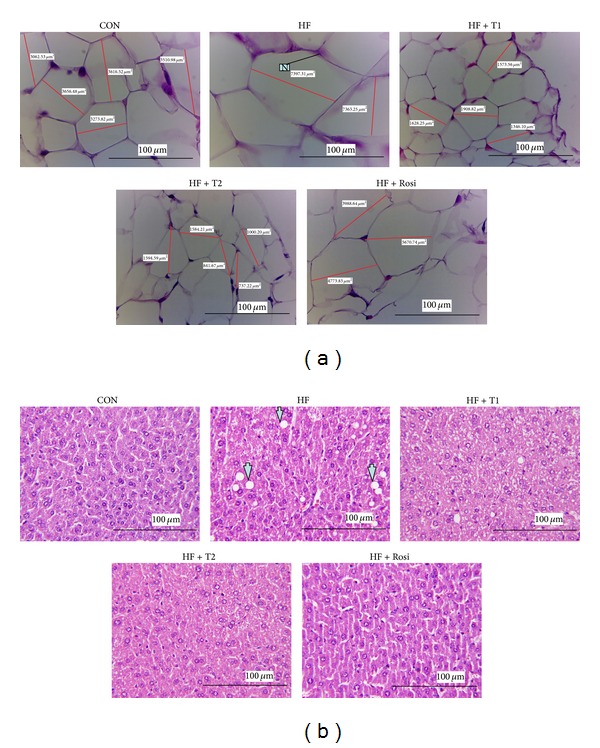
Effects of caffeamide** 36-13** (TS) on epididymal WAT and liver tissue morphology in the low-fat (CON), high-fat (HF), HF + T1, HF + T2, or HF + Rosi groups. Pictures of hematoxylin and eosin-stained sections of (a) mean area of adipocytes (*μ*m^2^) for epididymal WAT (magnification: 10 (ocular) ×20 (object lens)) from mice fed with TS. White adipose tissue (named adipocytes) is polyhedral by H&E stain, and the appearance showed string-like cytosol surrounding a vacuole. This is because of being embedded in paraffin as immersed in lipid solvents, and finally all the fats were removed. It was carefully observed unobvious nucleus (N) in the other side of cells; and (b) liver tissue (magnification: 10 (ocular) ×20 (object lens)) from mice fed with TS. The high-fat diet induced the hepatic ballooning degeneration in the HF group as compared with the CON group. The ballooning degeneration is a form of liver parenchymal cell death and the nucleolus was squeezed into the other side named balloon (as the arrow indicated). This may be due to the heap of glycogen in the nucleus. High-fat diet induced obesity and insulin resistance. Insulin levels affected the storage of hepatic glycogen. Treatment with T1 and T2 significantly decreased the degree of ballooning degeneration. Each presented is typical and representative of nine mice. Each presented is typical and representative of nine mice. TS:** **T1:** **10, T2:** **20 mg/kg bodyweight; Rosi: rosiglitazone (0.01 g/kg body weight).

As shown in Figure 3(a), feeding the HF diet induced hypertrophy of the adipocytes compared with the CON group in epididymal WAT. The average area of the cut of the adipocytes in the HF group (6765.87 *μ*m) is larger than in the CON group (3263.78 *μ*m). Treatment with T1 (1827.59 *μ*m) and T2 (1651.31 *μ*m) significantly decreased the hypertrophy compared with the HF group. The average area of the cut of the adipocytes in the Rosi-treated group is (4600.96 *μ*m). As shown in Figure 3(b), feeding the HF diet induced the ballooning of hepatocyte compared with the CON group in liver tissue. Afterwards, treatment with T1, T2, and Rosi decreased the ballooning compared with the HF group.

### 3.7. Expressions of PEPCK, G6Pase, FAS, Apolipoprotein C-III (apo C-III), and Adiponectin in Liver Tissue

**Figure 4:**
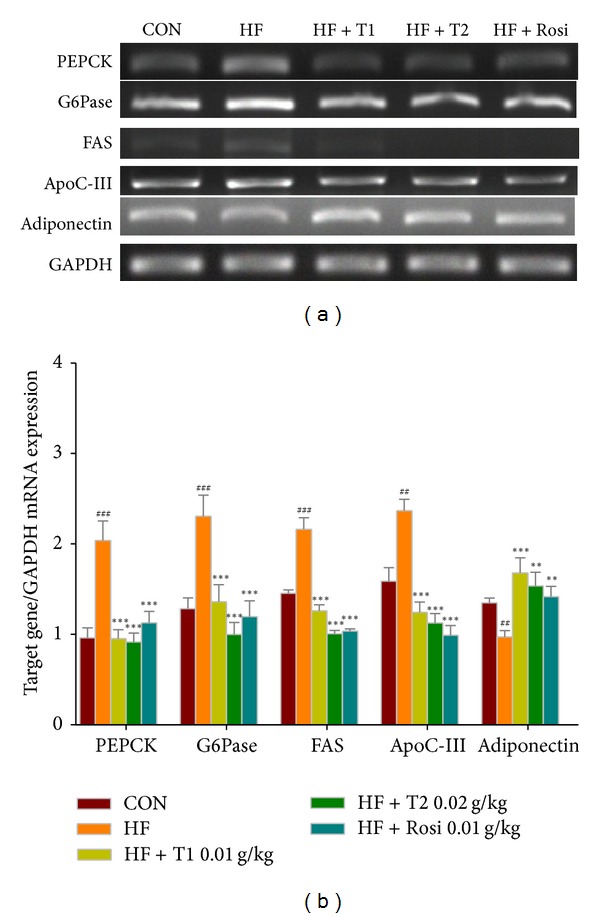
Semiquantitative RT-PCR analysis on PEPCK, G6Pase, SREBP1c, FAS, apo C-III, and adiponectin mRNA expression in liver tissue of the mice by oral gavage caffeamide** 36-13** (TS) for 4 weeks. All values are means ± S.E. (*n* = 9). ^##^ *P* < 0.001, ^###^ *P* < 0.001 compared with the control (CON) group; *P < 0.05, **P < 0.01, ***P < 0.001 compared with the high-fat + vehicle (HF) group. TS: T1: 10, T2: 20 mg/kg bodyweight; Rosi: rosiglitazone (0.01 g/kg body weight). Total RNA (1 *μ*g) isolated from tissue was reverse transcripted by MMLV-RT, and 10 *μ*L of RT products were used as templates for PCR. Signals were quantitated by image analysis; each value was normalized by GAPDH.

As shown in Figure 4, at week 12, the mRNA levels of PEPCK, G6Pase, FAS, and apo C-III were higher in the HF group than in the CON group (*P* < 0.001, *P* < 0.001, *P* < 0.001, and *P* < 0.01, resp.), whereas the expressions of adiponectin were lower in the HF group than in the CON group (*P* < 0.01). Following treatment, the T1-, T2-, and Rosi-treated groups decreased the mRNA level of PEPCK, G6Pase, FAS, and apo C-III, whereas increased the mRNA level of adiponectin as compared with the HF group (*P* < 0.001, *P* < 0.01, and *P* < 0.01, resp.).

### 3.8. The GLUT4 Protein Contents in Skeletal Muscle

**Figure 5:**
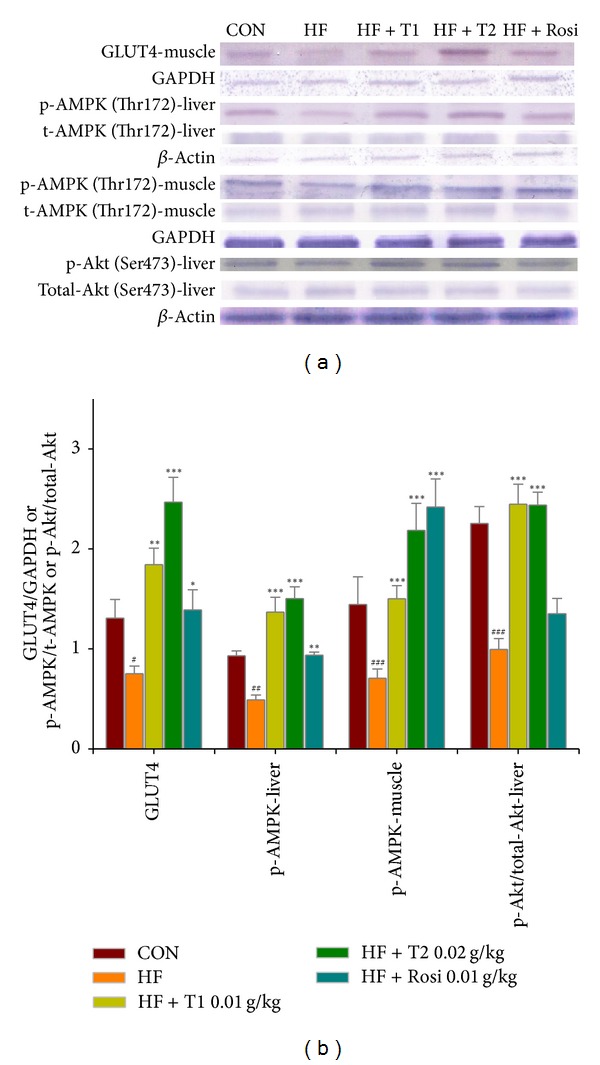
The protein contents of GLUT4 in skeletal muscle, the ratio of phospho-AMPK (Thr172) to total AMPK in liver tissue and skeletal muscle, and quantified results for the phosphorylation status of Akt (p-Akt normalized to total Akt (pAkt/Akt)) in liver tissue of the mice by oral gavage caffeamide** 36-13** (TS) for 4 weeks. Protein was separated by 12% SDS-PAGE detected by western blot. All values are means ± S.E. (*n* = 9). ^#^ *P* < 0.05, ^##^ *P* < 0.01, ^###^ *P* < 0.001 compared with the control (CON) group; *P < 0.05, **P < 0.01, ***P < 0.001 compared with the high-fat + vehicle (HF) group. TS:** **T1:** **10, T2: 20** **mg/kg bodyweight; Rosi: rosiglitazone (0.01 g/kg body weight).

As shown in Figure 5, at week 12, the protein contents of GLUT4 were lower in the HF group than in the CON group in skeletal muscle (*P* < 0.05). 4-week treatment resulted in increased GLUT4 protein contents in skeletal muscle in T1-, T2-, and Rosi-treated groups as compared with HF group (*P* < 0.01, *P* < 0.001, and *P* < 0.05, resp.).

### 3.9. The Phospho-AMPK (Thr172) Protein Contents in Liver Tissue and Skeletal Muscle

As shown in Figure 5, at week 12, the protein contents of phospho-AMPK protein were lower in the HF group than in the CON group in liver and skeletal muscle (*P* < 0.01, *P* < 0.001, resp.). After treatment, the protein contents of hepatic phospho-AMPK were increased in the T1-, T2-, and Rosi-treated groups compared with the HF group (*P* < 0.001, *P* < 0.001, and *P* < 0.01, resp.). Following treatment, the muscular protein contents of phospho-AMPK were increased in the T1-, T2-, and Rosi-treated groups compared with the HF group (*P* < 0.001, *P* < 0.001, and *P* < 0.001, resp.).

### 3.10. The Phospho-Akt (Ser 473)/Total Akt Protein Contents in Liver Tissue

As shown in Figure 5, at week 12, the protein contents of phospho-Akt/total-Akt were lower in the HF group than in the CON group in liver tissue (*P* < 0.001). 4-week treatment resulted in increased phospho-Akt/total-Akt protein contents in liver tissue in T1- and T2-treated groups as compared with HF group (*P* < 0.001, *P* < 0.001, resp.).

## 4. Discussion

The principle finding of this study was that TS has effectively lowered glycaemia and triglycerides which were involved in the peripheral tissues. In this study, we demonstrated that the AMPK and GLUT4 are instrumental in TS-mediated metabolic function. TS is one of caffeic acid amide derivatives; those structurally similar constituents (including KS370G and CAPE) have been shown to exert antihyperglycemic activity. The main difference between TS and KS370G is that the structure of TS is a piperidine ring. However, the antidiabetic and antihyperlipidaemic activity ofTS is not well defined.

The mouse model C57BL/6J mouse is susceptible to HF diet-induced not only marked increases in adipose tissue mass, but also pronounced insulin resistance, hyperlipidemia, and hyperinsulinemia [3, 25]. Therefore, we conducted our animal study using HF diet-induced diabetic states. The mouse C57BL/6 model fed with a HF diet could induce insulin resistance, obesity, hyperlipidemia, hyperinsulinemia, hyperleptinemia, and excess circulating free fatty acid [26, 27]. C57BL/6 mouse fed a HF diet is a robust and efficient model for early type II diabetes and therefore is used for both mechanistic studies and as a tool for developing novel therapeutic interventions [28]. Conversely, the Jackson Laboratory comments that mice do not develop overt diabetes when on high-fat diet (60%) versus low-fat diet (10%). It has been shown that hyperglycemia develops within 1 month of introduction of a HF diet in C57BL/6J mouse, and diabetes/obesity syndrome worsens with time and with increasing obesity; moreover, C57BL/6J mice fed a HF diet at 16 weeks had developed to the increased abdominal fat mass [25], and our study result at week 12 was in accordance with these. Consistent with others observations, feeding C57BL/6J mice with HF diet was able to induce hyperglycemia, obesity, hyperinsulinemia, hypertriglycemia, hyperleptinemia, and excess circulating free fatty acid [25–28]. Following treatment of HF-fed mice with TS, blood glucose concentration, circulating triglycerides, and visceral fat mass were lowered as well as a reduction in free fatty acid and improved insulin resistance.

The present study demonstrated that TS increased the protein contents of GLUT4 and had favorable effects on glucose uptake into the peripheral tissues and effectively lowered glycaemia. Furthermore, TS not only increased the phosphorylation of AMPK both in skeletal muscle and liver tissue, but also improved lipid metabolism. The AMPK activator AICAR has been shown to lower plasma glucose and ameliorate insulin resistance in animal studies [29, 30]. Based on our findings described, we found that TS may have favorable effects on glucose level and lipid metabolism. Further, TS is effective to improve insulin resistance and dyslipidemia in a mouse model of type 2 diabetes and dyslipidemia. These findings are involved in the results on GLUT4 contents and AMPK activation.

One of the difficulties in pursuing the mechanism of action of the compound at the present stage is its limited availability due to TS being a newly synthesized organic compound. Therefore, TS was firstly administered at the concentration of 0.04 g/kg in OGTT test by ICR mice. It was found that TS could lower glucose levels. Afterwards, TS was administered at the concentration of 0.01, 0.02 g/kg in chronic HF-fed mice. Based on the few amounts of TS and the duration of 4-week chronic treatment of experimental design and caffeamide derivative, KS370G at dosage of 10 mg/kg [11] exerting antihyperglycemic effect [11], the concentration of TS is lowered to 0.01, 0.02 g/kg in HF-fed mice; the daily dosage is expected to have an accumulative effect and long-lasting in effectiveness by the reason of TS being one of 3° amides not easy to cut off the structure; thus, its half life should be longer than the other derivatives and not easily to be metabolized.

To pursuing the mechanism of TS compound on antidiabetes, we chose GLUT4 translocation in skeletal muscle as the primary tissue, because skeletal muscle is the major tissue responsible for insulin-mediated glucose utilization [31]. This clarification measures the movement of the insulin responsive glucose transporter GLUT4 to the cell surface, which is an essential step for insulin-responsive glucose transport in muscle that becomes defective in insulin resistance [32]. In this study, the results showed that TS exhibited a strong effect to stimulate GLUT4 translocation in muscle to a level comparable with that of treatment with same doses of Rosi. The present results demonstrated that TS increased contents of GLUT4 resulting in increased glucose uptake by skeletal muscle thereby facilitating normoglycemia.

Since the phosphorylation of AMPK is another major regulator of GLUT4 translocation during exercise or in response to some antidiabetic agents such as AICAR or metformin [32], we investigated whether the AMPK phosphorylation is involved. We found that TS was able to increase the phosphorylation of AMPK in muscle to a relative level, suggesting that the AMPK phosphorylation is likely responsible for the improvements of GLUT4 contents by TS. Although it is not clear whether this is by direct or indirect stimulation, it remains to be further studied.

Lack of skeletal muscle AMPK*α*2 activity in transgenic mice exacerbates the development of diet-induced glucose intolerance and insulin resistance [33]. In contrast, activation of AMPK improves symptoms of impaired glucose homeostasis and insulin resistance [34–36]. We observed that TS was able to increase the phosphorylation of AMPK in skeletal muscle. The AMPK activator AICAR has been shown to lower plasma glucose and ameliorate insulin resistance in high-fat-fed rats [29]. Based on our findings described above, we predicted that TS may have similar effects on glucose metabolism in high-fat-fed mice.

Liver gluconeogenesis is known to account for approximately the majority of the hepatic glucose production [37]. PEPCK is a key rate-limiting enzyme of gluconeogenesis. The activities of the gluconeogenic enzymes also including glucose 6-phosphoatase (G-6Pase) were significantly increased in the liver of diabetic rats [38]. G-6Pase plays a vital role in glucose homeostasis [39]. Insulin integrates hepatic carbohydrate metabolism by increasing the biosynthesis of enzymes of glycolysis and glycogenesis and by inhibiting gluconeogenesis [40]. TS treatment reduced the expressions of these enzymes including PEPCK and G-6Pase. Therefore, downregulation of PEPCK and G-6Pase also contributes to the antidiabetic effect of TS. The decreased glucose concentration in HF-fed mice might be the results of restoration of these carbohydrate metabolism enzymes. Metformin activates AMPK and lowers blood glucose concentration by inhibiting hepatic glucose production and stimulating glucose disposal in skeletal muscle [41]. Liver phospho-AMPK proteins were increased in TS- and Rosi-treated groups. This might also indicate that TS has the ability to improve hyperglycemia through AMPK activities in gluconeogenesis.

In addition, Akt activation in liver contributes to the insulin-mediated suppression of gluconeogenesis. In this study we show that TS significantly enhanced Akt phosphorylation in liver in HF-fed mice. Coffee ingestion (contained in caffeic acids, etc.) is reported to enhance Akt phosphorylation in liver in diabetic KK-*A* ^y^ mice [42]. Therefore, it is possible that TS caused glucose lowering by not only AMPK phosphorylation but also hepatic Akt phosphorylation, thus inhibiting hepatic glucose production via PEPCK and G6Pase downregulation. Certainly, the stimulation of glucose disposal in skeletal muscle by TS might also contribute to the hypoglycemic effect.

AMPK has been shown to regulate a variety of different metabolic pathways; thus it is recognized as a useful target for the treatment of metabolic disorders including T2D and dyslipidemia [30, 43]. Activation of AMPK has been shown to increase fatty acid oxidation, whereas it inhibits lipid synthesis and could improve insulin action [29, 30]. For this reason, the second objective of this study was to look into the antihyperlipidemic effect of TS. PPAR*α* ligands also increase the expression of the lipoprotein lipase gene and reduce the expression of the apo C-III gene, which is an inhibitor of lipoprotein lipase [44], thus resulting in hypotriglyceridemic effect. Our results suggest that TS acts by apo C-III and improves plasma lipid profiles. In addition, following treatment with TS, triglycerides lowering occurred as a result of downregulation of the enzyme, FAS. The AMPK activator metformin has been shown to downregulate the FAS expression through AMPK activation [20]. Therefore, it is possible that TS inactivated these enzymes and/or downregulated gene expressions through AMPK activation.

In this study WAT histology has revealed that the number of large adipocytes was decreased while the number of small adipocytes was increased by TS. Thus, we think TS may be able to mobilize fat from adipose tissue by increasing lipid catabolism in liver, because lipids that accumulate in adipose tissue are largely derived from circulating TG [45] and liver is a major target tissue for lipid and lipoprotein metabolism. Based on our results, TS possibly decreased the TG synthesis in liver which effectively regulated morphometric adipocytes.

Treatment of rat adipocytes with globular domain of adiponectin is reported to increase in the glucose uptake and AMPK activation [46]. Our findings show that TS significantly increased AMPK activation and adiponectin levels. Hypoadiponectinemia appears to play an important and causal role in insulin resistance, type 2 diabetes, and metabolic syndrome [47]. An increase in adiponectin levels or gene expressions is reported to be beneficial for insulin resistance [47, 48]. Therefore, TS could provide a therapeutic advantage to improve insulin resistance. In addition, Minokoshi et al. [31] demonstrated that leptin activated AMPK, and this activation is strongly associated with the suppression of hepatic lipid accumulation. Considering the two different mechanisms of AMPK activation of this compound, it is possible that TS could directly activate AMPK or affect the secretion or gene expression of adiponectin and leptin by inducing AMPK activation. The target molecule for TS should be identified.

In conclusion, this study revealed that TS showed protective effects on diabetes and hyperlipidemia in HF-fed mice, and such effect might be associated with increased protein contents of GLUT4 and increased phosphorylation of AMPK both in liver and skeletal muscle. TS improved glycemic control primarily by increased contents of muscular GLUT4 to elevate glucose uptake; on the other hand, suppression of hepatic glucose production (downregulation of PEPCK and G-6Pase). Moreover, TS increased hepatic phosphorylation of AMPK while suppressing lipogenic enzyme expression (including FAS), thus contributing to the lowering of circulating triglycerides. Therefore, TS may be beneficial for the management of type 2 diabetes and hypertriglyceridemia.

## Figures and Tables

**Table 1:** Primers used in this study.

Gene	Accession numbers	Forward primer and reverse primer	PCR product (bp)	Annealing temperature (°C)
Liver				
PEPCK	NM_011044.2	F: CTACAACTTCGGCAAATACC R: TCCAGATACCTGTCGATCTC	330	52
G6Pase	NM_008061.3	F: GAACAACTAAAGCCTCTGAAAC R: TTGCTCGATACATAAAACACTC	350	50
FAS	NM_007988	F: TGGAAAGATAACTGGGTGAC R: TGCTGTCGTCTGTAGTCTTG	240	50
apo C-III	NM_023114.3	F: CAGTTTTATCCCTAGAAGCA R: TCTCACGACTCAATAGCTG	349	47
Adiponectin	NM_009605.4	F:TCTTCTACAACCAACAGAATCA R:GTATCATGGTAGAGAAGGAAGC	324	50.5
GAPDH	NM_031144	F: TGTGTCCGTCGTGGATCTGA R: CCTGCTTCACCACCTTCTTGA	99	55

**Table 2:** Effects of caffeamide **36-13** (TS) on absolute tissue weight, food intake, liver lipids and blood profiles.

Parameter	CON	HF	HF + T1	HF + T2	HF + Rosi
			0.01^a^	0.02^a^	0.01^a^
Absolute tissue weight (g)					
EWAT	0.349 ± 0.003	1.481 ± 0.158^###^	0.999 ± 0.105*	0.832 ± 0.082***	1.003 ± 0.103*
MWAT	0.278 ± 0.014	0.518 ± 0.070^###^	0.406 ± 0.016**	0.372 ± 0.025***	0.238 ± 0.033***
RWAT	0.087 ± 0.010	0.671 ± 0.059^###^	0.472 ± 0.065*	0.373 ± 0.050**	0.334 ± 0.057*
Visveral fat	0.429 ± 0.027	2.148 ± 0.198^###^	1.471 ± 0.166**	1.205 ± 0.130***	1.330 ± 0.138***
BAT	0.127 ± 0.025	0.191 ± 0.023	0.162 ± 0.042	0.121 ± 0.014*	0.210 ± 0.023
Liver (g)	0.906 ± 0.035	1.078 ± 0.075	0.999 ± 0.060	1.030 ± 0.117	0.871 ± 0.075
Spleen	0.059 ± 0.003	0.075 ± 0.004	0.071 ± 0.003	0.074 ± 0.006	0.080 ± 0.005
Weight gain (g)	−0.12 ± 0.31	2.61 ± 1.04^##^	1.71 ± 0.59	0.43 ± 0.59	0.91 ± 1.45
Food intake (g/day/mouse)	2.01 ± 0.02	1.73 ± 0.05^###^	1.62 ± 0.04	1.62 ± 0.05	1.74 ± 0.05
Liver lipids					
total lipid (mg/g)	51.0 ± 3.6	91.1 ± 5.2^###^	69.8 ± 3.7*	60.2 ± 5.9**	60.2 ± 5.4**
Triacylglycerol (*μ*mol/g)	30.1 ± 3.3	74.4 ± 6.8^###^	53.6 ± 5.6*	46.1 ± 6.2**	42.6 ± 5.8**
Blood profiles					
Glucose levels (mg/dL)	84 ± 5.9	142.3 ± 7.1^###^	98.5 ± 8.4**	98.0 ± 7.6**	88.5 ± 5.8***
Triglycerides (mg/dL)	56.4 ± 4.1	107.4 ± 5.8^###^	77.5 ± 5.8**	75.9 ± 4.7***	81.8 ± 5.8*
FFA (meq/L)	1.64 ± 0.08	2.68 ± 0.07^##^	1.32 ± 0.14**	1.27 ± 0.09***	1.52 ± 0.16*
TC (mg/dL)	91.0 ± 4.7	183.4 ± 6.8^###^	175.0 ± 7.0	184.9 ± 23.8	134.9 ± 3.5
Insulin (*μ*U/mL)	20.13 ± 5.88	151.03 ± 15.53^###^	53.44 ± 13.85*	47.45 ± 10.29**	51.10 ± 19.97*
Adiponectin (ng/mL)	7.12 ± 0.31	4.39 ± 0.22^##^	6.72 ± 0.37*	7.35 ± 0.49**	7.51 ± 0.53**
Leptin (*μ*g/mL)	1.230 ± 0.062	2.923 ± 0.056^###^	2.155 ± 0.100***	2.009 ± 0.105***	2.063 ± 0.111***

All values are means ± S.E. (*n* = 9). ^#^ *P* < 0.05, ^##^ *P* < 0.01, ^###^ *P* < 0.001 compared with the control (CON) group; *P < 0.05, **P < 0.01, ***P < 0.001 compared with the high-fat + vehicle (distilled water) (HF) group. TS: T1: 0.01, T2: 0.02 g/kg bodyweight; Rosi: rosiglitazone (0.01 g/kg body weight). BAT, brown adipose tissue; RWAT, retroperioneal white adipose tissue; MWAT, mesenteric white adipose tissue; FFA, plasma free fatty acid; TC, total cholesterol; TG, triglyceride. ^a^Dose (g/kg/day).

## Abbreviations

AMPK: AMP-activated protein kinase
apo C-III: Apolipoprotein C-III
BAT: Brown adipose tissue
CON: Control
EWAT: Epididymal white adipose tissue
FFA: Free fatty acid
GAPDH: Glyceraldehyde-3-phosphate dehydrogenase
G6Pase: Glucose-6-phosphatase
GLUT4: Glucose transporter 4
HF: High-fat control
MWAT: Mesenteric white adipose tissue
PPAR*α*: Peroxisome proliferator-activated receptor *α*
Rosi: Rosiglitazone
RT-PCR: Reverse transcription-polymerase chain reaction
RWAT: Retroperitoneal white adipose tissue
TC: Total cholesterol
TG: Triglyceride
WATs: White adipose tissues.
